# A Case Study on Fish Gelatin/Microcrystalline Cellulose Biomaterial Inks for Extrusion-Based Bioprinting

**DOI:** 10.3390/gels11060458

**Published:** 2025-06-16

**Authors:** Yubo Tao, Jinbao Du, Tong Hu, Peng Li, Ling Pan, Fangong Kong, Jingfa Zhang

**Affiliations:** 1 State Key Laboratory of Green Papermaking and Resource Recycling, Qilu University of Technology (Shandong Academy of Sciences), Jinan 250353, China; taoyubo@qlu.edu.cn (Y.T.); hey13383927335@163.com (T.H.); kfgwsj1566@163.com (F.K.); 2 Key Laboratory of Bio-Based Material Science & Technology, Northeast Forestry University, Harbin 150040, China; 3 School of Art and Design, Wuhan Polytechnic University, Wuhan 430024, China; pl_3195@163.com

**Keywords:** fish gelatin, microcrystalline cellulose, extrusion bioprinting, biomaterial ink

## Abstract

The development of printable, biocompatible, biodegradable, and cost-effective bioinks, or biomaterial inks, remains a focal point in extrusion-based bioprinting research. In this study, fish gelatin (FG) was reinforced with microcrystalline cellulose (MCC) to formulate biomaterial inks. These FG/MCC composite inks were fabricated into 3D scaffolds using an extrusion bioprinter. The influence of MCC concentration on printability was systematically evaluated. Fourier transform infrared spectroscopy (FTIR) and X-ray diffraction (XRD) analyses confirmed the formation of hydrogen bonds between MCC and FG, indicating molecular-level interactions. Notably, MCC incorporation enhanced the rheological properties of the ink and significantly improved the compressive strength of printed scaffolds. Furthermore, MCC content modulated key scaffold characteristics, including porosity, degradation rate, swelling behavior, and microarchitecture. These findings demonstrate that FG/MCC composite hydrogels exhibit optimal properties for extrusion-based 3D bioprinting, offering a promising platform for tissue engineering applications.

## 1. Introduction

One of the most popular bioprinting techniques, extrusion-based bioprinting, is commonly used in fabrication of tissue-like constructs due to its affordability and easy of maneuverability [1]. In this method, bioinks/biomaterial inks [2] are first loaded into a printhead. Under pneumatic or mechanical pressure, the material extrudes from the printhead in the form of filaments, which are deposited along designated paths in a layer-by-layer manner to build the desired 3D constructs [3]. Developing printable bioinks/biomaterial inks that are biocompatible, biodegradable, and cost-efficient is an active area of research [4].

Gelatin is derived from the partial hydrolysis of fibrous protein collagen, which is the principal constituent of animal skin, bone, and connective tissue [5]. The triple-helical structure of collagen molecules consists of three counterclockwise-rotated α-chains [6]. Common treatments during gelatin manufacturing—such as exposure to diluted acid or alkali—result in partial cleavage of the triple-helix [7]. Gelatin forms once sufficient structural integrity is lost, rendering the material soluble in warm water [6]. Due to its biocompatibility, biodegradability, and low antigenicity [8], gelatin is widely used in food [9], medicine [10], and tissue engineering [11]. Notably, gelatin-based bioinks have drawn increasing attention in extrusion bioprinting because of their structural similarity to the extracellular matrix (ECM) [8]. Traditionally, gelatin is sourced from terrestrial mammals (e.g., pigs and cows). However, concerns over prion diseases and religious restrictions have driven the development of fish gelatin (FG) as a preferred alternative [12,13]. Additionally, FG production adds value to fish processing byproducts—such as skin, bones, fins, and intestines—that would otherwise be discarded [14]. Consequently, FG has gained significant research interest in recent years.

Cellulose, an abundant and renewable natural biopolymer, consists of linear chains comprising several thousand β (1–4)-linked D-glucose units [15]. Its semi-crystalline structure arises from a combination of amorphous and ordered regions [16], where glucose chains associate through intermolecular/intramolecular hydrogen bonds and van der Waals forces. By selectively degrading the amorphous domains via chemical or physicochemical treatments, the crystalline components can be isolated to yield functional materials at either the microscale (microcrystalline cellulose, MCC) or nanoscale (nanocrystalline cellulose, NCC) [17]. Compared with NCC, MCC has a processing cost advantage. Additionally, previous studies have shown that when preparing films via the casting method using all-cellulose-based materials, MCC films exhibit higher tensile strength and modulus than NCC films [18]. In nanocomposite materials, MCC is an increasingly favored reinforcing agent due to its high specific surface area, good biocompatibility (Figure S1), biodegradability, nontoxicity (Figure S2) and renewability, as well as strong mechanical properties [19]. MCC has been found to have great potential as an additive for reinforcing composite hydrogels [20]. The existing literature indicates that MCC-reinforced FG composite films exhibited enhanced mechanical properties, thermal stability, and moisture resistance [21]. Moreover, MCC was found to have good interfacial compatibility with the FG matrix, as demonstrated in recent studies [22].

With increasing attention paid to the field of bioprinting, various studies have explored the effects of additives on the biomaterial inks used in extrusion bioprinting. Specifically, additives such as oxidized alginate [23], graphene oxide [24], and silk fibroin [25] have been found to improve the overall printability of gelatin-based bioinks. Nanocellulose-fibril-reinforced gelatin composite hydrogels can exhibit shape fidelity, structural integrity, and compressive resistance that meet the requirements for printing tasks [26,27]. A bioink formulation consisting of pectin, carboxymethyl cellulose (CMC), and MCC has been developed through recent research. The resulting pectin–MCC composite bioink demonstrated optimal printability characteristics, making it particularly suitable for applications in personalized wound dressing fabrication [28]. Nevertheless, no studies covering the development and utilization of FG/MCC biomaterial inks for the 3D bioprinting of scaffold structures were found during the literature review. This study explores the feasibility of FG/MCC composite hydrogels as biomaterial inks. The effects of MCC content on the printability and properties of the biomaterial ink were investigated. Overall, this work serves as a preliminary investigation into the viability of FG/MCC composite hydrogels as a candidate biomaterial ink for extrusion bioprinting applications.

## 2. Results and Discussion

### 2.1. Printability

The printability of FG/MCC composite hydrogels was assessed through initial screening, qualitative characterization, and rheological evaluation.

#### 2.1.1. Printing Temperature

**Figure 1:**
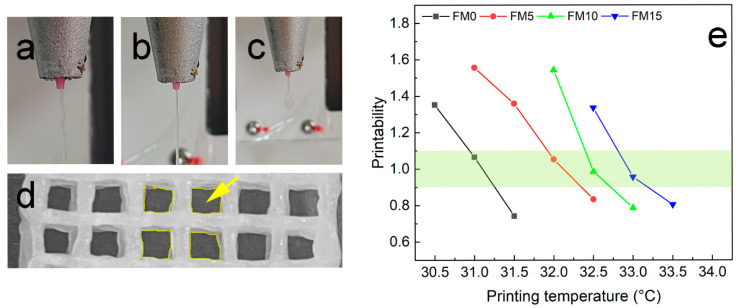
Evaluation the printability of the FG/MCC biomaterial inks: (**a**–**c**) shapes of extrudes; (**d**) pores were selected for calculating $P_{r}$; (**e**) qualitative characterization of printability.

Gelatin-based biomaterial inks have narrow printing temperature windows [8]. Hence, setting an optimal print temperature is critical for the printability of such materials. As shown in Figure 1a, the high viscosity of ink at temperatures lower than optimal would result in irregular filament extrusion. If the temperature is above the optimal range, the viscosity of the ink would become too low for stable extrusion. This phenomenon can be demonstrated in Figure 1c, where the extrudate exhibits liquid-like and uncontrollable behavior. Conversely, within the optimal temperature range, the biomaterial inks could be successfully extruded with consistent quality, as shown in Figure 1b. This method provides a preliminary screening approach to determine the suitable printing temperature range.

An algorithm based on pore geometry in the fabricated constructs was employed for qualitative printability characterization [29], expressed as

(1) $$P_{r}=\frac{L^{2}}{16A}$$

where $P_{r}$ is the printability, L is the perimeter, and A is the area of the pore. Materials with good printability should achieve a $P_{r}$ value between 0.9 and 1.1 [8]. As shown in Figure 1d, nine pores were analyzed to determine the mean value of $P_{r}$. Figure 1e demonstrates the $P_{r}$ values of FG/MCC biomaterial inks across different printing temperatures. With the increase of MCC content, the optimal printing temperature gradually increases. Data points within the light yellow-green colored region (0.9 < $P_{r}$ < 1.1) indicate the suitable printing temperature for FG/MCC biomaterial inks. Excessive MCC content (e.g., the FM20 sample) compromises printability, leading to extrusion failure.

#### 2.1.2. Rheological Properties

**Figure 2:**
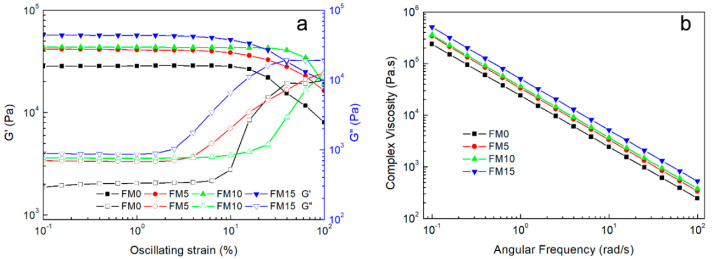
(**a**) Storage modulus (G′) and loss modulus variation (G″) and (**b**) shear-thinning behavior.

The linear viscoelastic region (LVR), defined by a critical strain or stress threshold at its boundary, is typically identified through amplitude sweep tests. In these tests, the oscillating strain is incrementally increased while maintaining a constant frequency [30]. As shown in Figure 2a, the storage modulus (G′) of most samples remained stable until a rapid decline occurred at approximately 10% oscillation strain, except for FM10, which maintained its G′ until ~20% strain. The inflection point of each curve represents the critical oscillation strain, delineating the LVR boundary [31].

At 40–50% oscillation strain, the crossover between the storage modulus (G′) and loss modulus (G″) marks the critical transition point where the hydrogel shifts between gel and sol states. When G′ > G″, the FM/MCC inks exhibited gel-like behavior with sufficient stability for printing, whereas G′ < G″ resulted in sol-like composites that failed to maintain structural definition post-extrusion [29].

Additionally, the G′ and G″ values provide insights into the hydrogel’s mechanical properties. Both moduli showed a positive correlation with MCC content, which can be attributed to hydrogen bonding between MCC and FG enhancing the hydrogel’s elasticity and stability [32,33].

The complex viscosity of the composite hydrogels with varying MCC content is shown in Figure 2b. As the angular frequency increases, the complex viscosity of the hydrogel composite gradually decreases, exhibiting shear-thinning behavior. This rheological property was characterized by fitting the Power Law model to the linear region of the shear rate–viscosity curve [34].

(2) $$\eta =k{\left(\dot{\gamma }\right)}^{n-1}$$

where η is the viscosity, $\dot{\gamma }$ is the shear rate, k is the consistency index (flow coefficient), and n describes the shear-thinning properties of the sample. According to the Cox–Merz rule, the curves of the complex viscosity modulus versus angular frequency and shear viscosity versus shear rate coincide [35]. The calculated *n* < 1 confirms the shear-thinning behavior, making the hydrogel composite suitable for extrusion-based printing [36].

### 2.2. Scaffold Properties

#### 2.2.1. Morphology

**Figure 3:**
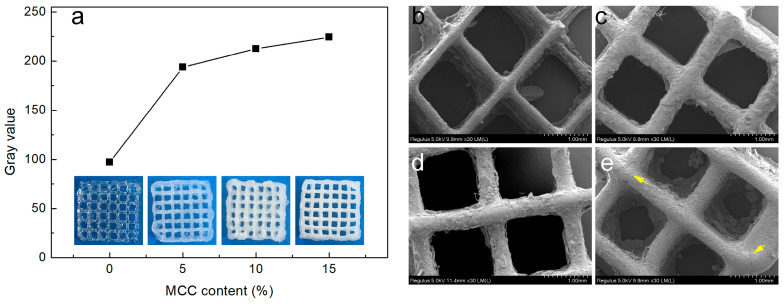
(**a**) Macroscopic morphology and gray values of printed scaffolds; (**b**–**e**): SEM images of the printed scaffolds (FM0, FM5, FM10, and FM15, respectively).

The macroscopic morphology of the printed composite hydrogel scaffold is shown in Figure 3a. The transparent hydrogel exhibits a transition to white opacity (with increased gray values) as MCC content rises. The printing stability and structural consistency of the composite hydrogels are significantly enhanced by MCC incorporation, as confirmed by visual inspection of the printed samples.

SEM analysis revealed the microstructural effects of MCC additives on the printed scaffold. As shown in Figure 3b–e, minor phase separation between MCC and FG was observed, primarily attributed to pore formation during freeze-drying-induced water sublimation. Notably, microcracks (yellow arrow, Figure 3e) appeared in the FM15 sample (highest MCC content), suggesting incomplete dispersion of excessive MCC fillers within the FG matrix. Agglomerated sites likely compromised the composite hydrogel’s structural stability [22].

#### 2.2.2. Porosity and Swelling Ratio

**Figure 4:**
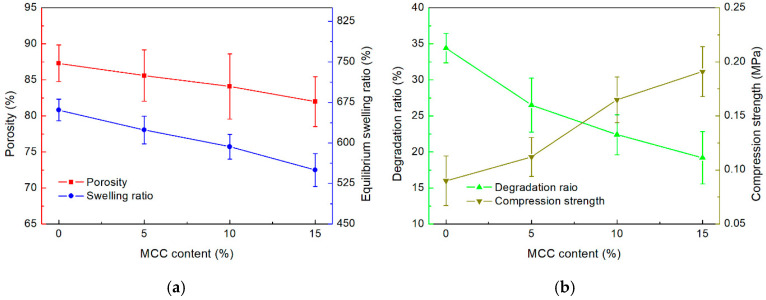
(**a**) Porosity and equilibrium swelling ratio; and (**b**) degradation ratio and compression strength of the printed composite scaffold.

The porous structure of scaffolds facilitates cell migration, while high porosity enhances the available surface area for cell adhesion, scaffold integration, and tissue interactions within a given volume [37]. The porosity of each FG/MCC composite scaffold is shown in Figure 4a. Evidently, the porosity decreased with increasing MCC content. The introduction of MCC altered the pore structure of the composite sponge. MCC filled the gaps between FG molecules, resulting in reduced void size within the FG matrix. Additionally, the interfacial interaction between MCC and the FG matrix promoted the cross-linking and aggregation of FG molecules, leading to a tighter network structure. This observation was confirmed by the FTIR results [21]. A scaffold porosity greater than 80% is considered high porosity, effectively enhancing biocompatibility [38]. Hence, the FG/MCC composite scaffold exhibits sufficient porosity (80–90%) for potential tissue engineering applications.

The swelling ratio of the scaffolds is an important factor in bioprinting applications. A swelling ratio larger than the suitable range will result in a bloated and mechanically weak scaffold structure. Meanwhile, a swelling ratio below the suitable range would reduce the scaffolds’ hydrophilicity and moisture content. The equilibrium swelling ratio ($S_{eq}$) of each scaffold sample is shown in Figure 4a. Specifically, the $S_{eq}$ of FM0 was measured to be the largest at 661%. Notably, the $S_{eq}$ decreased with the increase of MCC content. The sample with the largest MCC content, FM15, was found to have a $S_{eq}$ close to 550%. This phenomenon could be attributed to the formation of hydrogen bonds between MCC and FG, which disturbed the internal structure of FG. The non-uniform structure led to the decrease in structural stability and swelling performance of the FG/MCC composite [39].

#### 2.2.3. Degradation Ratio and Compression Strength

The stability of the printed scaffold is crucial for bioprinting applications. The degradation ratios of the printed scaffolds with different MCC contents are shown in Figure 4b. Evidently, the introduction of MCC fillers decreased the degradation ratios of the scaffolds. After 4 weeks of the experiment, the average degradation ratios recorded for FM0, FM5, FM10, and FM15 were 34.4%, 26.5%, 22.4%, and 19.2%, respectively. Notably, the pure FG scaffold collapsed after approximately 3 weeks. Hence, the addition of MCC enhanced the overall structural integrity and degradation resistance of the printed scaffolds.

**Figure 5:**
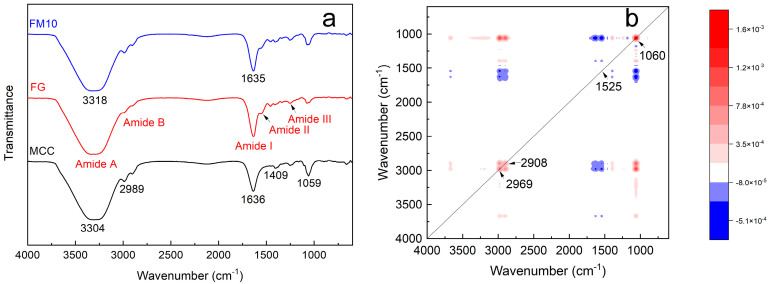
(**a**) FTIR of FG, MCC, and the FM10 hydrogel composite and (**b**) 2D synchronous correlation spectroscopy of FG and FM10.

The compression strength of scaffolds with different MCC contents are shown in Figure 4b. The compression strength of the printed scaffold increased with the addition of MCC. For instance, 15% MCC content resulted in a substantial increase in the average compression strength of the scaffold from 0.091 MPa (FM0) to 0.192 MPa (FM15). This increase could be attributed to the rigid network formed by MCC and the strengthening effect of hydrogen bonds formed between MCC and the FG matrix [32]. Notably, the reinforcement effect of MCC additives appears to only take effect at sufficient concentrations. As evident in Figure 5b, while a 5% MCC content only improved the compression strength marginally, a significant improvement was observed in samples with 10% MCC content. Moreover, excessive MCC content appears to have diminishing returns in improving the compression strength. Overall, it can be concluded that the introduction of MCC fillers could improve the compression strength of printed scaffolds [26].

### 2.3. Properties of the FG/MCC Composite Biomaterial Ink

#### 2.3.1. Fourier-Transform Infrared Spectroscopy (FTIR)

Figure 5a shows the FTIR spectra of the FG, MCC, and FM10 samples. The FTIR spectrum of FG mainly consists of five characteristic regions (amide-A, amide-B, amide I, amide II, and amide III) in the range of 400–4000 cm^−1^ [40]. The absorption peak at 3322 cm^−1^ corresponds to the amide-A band, whose properties (3200–3600 cm^−1^) are related to the N–H stretching vibration. The absorbance of amide-B appears at 2992 and 2905 cm^–1^, associated with asymmetric C–H and –NH_3^+^_ tensile vibrations (2900–3100 cm^−1^) [40,41]. The amide I band at 1634 cm^−1^ arises from C=O stretching and C–N stretching vibrations (1600–1700 cm^−1^). The amide II band (1500–1550 cm^−1^), observed near 1540 cm^−1^, results from N–H bending and C–N stretching vibrations. The amide III band (1200–1300 cm^−1^) at 1250 cm^−1^ primarily reflects C–N stretching, N–H bending, and –CH_2_ swing vibrations of the glycine main chain and proline side chain. Lastly, the peak at 1074 cm^−1^ is attributed to C–O stretching vibrations [40,42,43].

In the FTIR spectrum of MCC, peaks at 4000–2995 cm^−1^, 2989 cm^−1^, 1636 cm^−1^, 1409 cm^−1^, and 1059 cm^−1^ indicate the presence of crystal and amorphous regions [44]. The spectrum of the pure microcrystalline cellulose shows a strong, broad band at 3304 cm^−1^ and a band at 1636 cm^−1^, corresponding to the stretching and bending modes of surface hydroxyls. The peak at 2905 cm^−1^ is assigned to symmetric/asymmetric C-H stretching vibrations, while the absorption peak at 1059 cm^−1^ can be attributed to the C-O bonds in cellulose. The peak at 1409 cm^−1^ results from methylene deformation vibrations [45].

In the infrared spectrum of FM10 hydrogel composite, the absorption peak at 3318 cm^−1^ lies between those of the spectra of FG and MCC. This is likely the result of the superposition of –NH_2_ and –OH peaks from both components. Other characteristic peaks remain largely similar. In the two-dimensional synchronous correlation spectroscopy of FG and FM10 (Figure 5b), four auto-peaks are observed at 2969 cm^−1^, 2908 cm^−1^, 1525 cm^−1^, and 1060 cm^−1^. The auto-peak intensities of 2969 cm^−1^, 2908 cm^−1^, and 1060 cm^−1^ are significantly higher. Their positive cross-peaks indicate an increase in the number of functional groups, primarily due to the hydrogen bonding between FG and MCC. The enhanced structural strength of the 1060 cm^−1^ glucose ring vibration further confirms the effect of MCC incorporation into the FG system [21].

#### 2.3.2. X-Ray Diffraction Analysis (XRD)

**Figure 6:**
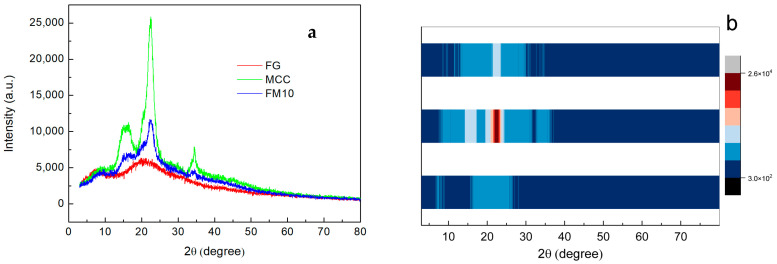
(**a**) XRD pattern and (**b**) XRD strip heat map of FG, MCC, and the FM10 hydrogel composite.

XRD analysis was used to examine the crystal structure and crystallinity of samples. The XRD patterns of FG, MCC, and FM10 are shown in Figure 6a. The XRD pattern of MCC shows typical diffraction peaks at 2θ ≈ 15°, 16°, 23°, and 34°, corresponding to the (101), (101̅), (002), and (040) crystal planes, respectively [46,47].

The XRD pattern of FG exhibits two diffraction peaks at 7° (peak 1) and 21° (peak 2), representing a triple helix structure and a single-handed helix chain, respectively. Specifically, the position of peak 1 correlates with the diameter of the triple helix, while its intensity reflects the triple helix content. Peak 2 corresponds to the inter-residue distance along the helix chain [41]. In the FM10 composite, both the characteristic MCC peaks and the broad FG diffraction peak are observed. The superposition of MCC and FG XRD patterns, combined with the absence of additional polymer peaks, suggests good compatibility between FG and MCC in the composite [48].

In Figure 6b, the addition of MCC causes a significant shift in the FG’s XRD peak from 7° to 10°. This shift likely results from intermolecular interactions between MCC hydroxyl groups and gelatin NH_2_ side chain groups. Such interactions limit molecular mobility, thereby hindering crystallization [41,48]. The results confirm hydrogen bond formation between MCC and FG, consistent with the FTIR data.

## 3. Conclusions

This study investigated the development of biomaterial inks for extrusion-based bioprinting utilizing fish gelatin derived from fish skin as the hydrogel matrix and microcrystalline cellulose as the reinforcement additive. Scaffold structures were successfully 3D printed using FG/MCC biomaterial inks with varying MCC content. Based on experiment results, the following can be concluded:

(1) FG/MCC biomaterial inks with varying MCC content require different optimal printing temperatures. However, the ink containing 20% MCC cannot be successfully printed.
(2) Rheological analysis demonstrated that an appropriate MCC content enhances the printability of FG/MCC biomaterial inks, while excessive MCC content leads to unnecessarily high viscosity, thereby impairing printability.
(3) MCC addition reduced the scaffold porosity and swelling ratio while improving the compressive strength. Importantly, MCC did not compromise the potential of FG-based inks for tissue engineering applications.
(4) FTIR and XRD results confirmed that MCC forms molecular interactions with the FG matrix.

In summary, the FG/MCC biomaterial inks demonstrate a promising biomaterial ink candidate suitable for extrusion bioprinting in fields such as tissue engineering and biomedical applications.

## 4. Materials and Methods

### 4.1. Materials

The fish skin of *Oreochromis niloticus* was purchased from Baiwei Biology Science and Technology Co., Ltd. (Guangdong, China). The MCC (α-Cellulose (C_6_H_10_O_5_)_n_, CAS 9004-34-6, Molecular Weight 162.06, Particle Size < 25 microns) was supplied by Aladdin Industrial Co., Ltd. (Shanghai, China). Sodium hydroxide (NaOH, AR), PBS solution, acetic acid (CH_3_COOH, AR), and glutaraldehyde grade II solution (25 wt.% in H_2_O) were supplied by Macklin Biochemical Co., Ltd. (Shanghai, China).

### 4.2. Preparation of FG/MCC Biomaterial Inks

**Fish gelatin extraction protocol:** Fish skin was first cut into 1 cm × 1 cm squares and washed with deionized water, followed by soaking in the 0.3 mol/L NaOH solution for 30 min. The alkali-treated fish skin was then rinsed with deionized water until achieving a neutral pH (7.0). Subsequently, the fish skin was submerged in the 0.15 mol/L CH_3_COOH solution for 20 min, and rewashed to neutrality. The pretreated fish skin underwent hydrolysis in deionized water at 60 °C for 6 h. The resulting mixture was pre-frozen at −20 °C for 24 h before freeze-drying for 72 h at −45 °C. Finally, the lyophilized product was ground into FG powder.

**Preparation of FG/MCC composite hydrogels:** First, FG powder was dissolved in deionized water at 50 °C for 40 min to prepare a 20% (*w*/*v*) FG solution. Preliminary experiments indicated that MCC content significantly affects the composite hydrogel’s viscosity. Insufficient viscosity leads to poor shape fidelity of printed constructs, whereas excessive viscosity hinders extrusion. Therefore, an optimal MCC concentration range of 5–20% (*w*/*w*, MCC/FG) was selected. Subsequently, MCC was added to the FG solution at 5%, 10%, 15%, and 20% (*w*/*w*) to form experimental groups, with neat FG serving as the control. The resulting biomaterial inks were labeled FM0 (control), FM5, FM10, FM15, and FM20 according to their MCC content. Finally, each mixture was processed in an ultrasonic cell crusher (JY98-iiiDN, Shanghai Huxi Industry Co., Ltd., Shanghai, China) for 5 min, followed by homogenization via magnetic stirring (2 h, 500 rpm).

### 4.3. 3D Printing of Scaffolds

**Figure 7:**
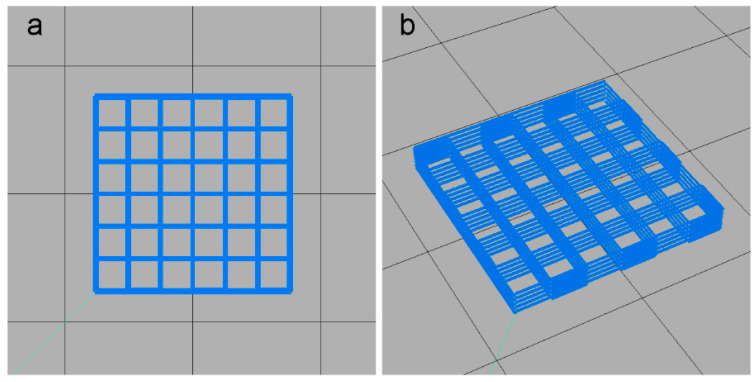
Digital model of the printed cubic scaffold. (**a**) Top view, and (**b**) perspective view.

An EFL-BP6601 extrusion printer (Yongqinquan Intelligent Equipment Co., Ltd., Suzhou, China) was used to fabricate a 15 mm × 15 mm × 3 mm scaffold model, as shown in Figure 7. The printing parameters were configured as follows: print bed temperature at 4 °C, printing speed at 6 mm/s, nozzle diameter at 0.26 mm, nozzle height at 0.2 mm, layer height at 0.2 mm, and line center spacing at 2.5 mm. The MCC/FG composite hydrogels were loaded into the printer barrel at 30 °C. The barrel temperature (printing temperature) was gradually increased at 0.5 °C per 10 min interval. The optimal printing temperature was determined by real-time monitoring of the filament morphology and qualitative analysis of the printed structures. Finally, scaffolds were pre-frozen at −20 °C for 24 h and lyophilized at −45 °C for 72 h to complete sample preparation.

### 4.4. Characterization of Biomaterial Ink

**Rheological testing:** A rotational rheometer (ARES/G2, TA Instruments, New Castle, DE, USA) was used to conduct amplitude sweep scanning on FG/MCC composite hydrogels at room temperature (25 ± 1 °C) to determine their linear viscoelastic range (LVR). The amplitude sweep was performed at a fixed frequency of 1 Hz with strain ranging from 0.1% to 1000%. Subsequently, frequency sweep tests were conducted within the established LVR (fixed strain: 1%) using an angular frequency range of 0.1–100 rad/s to evaluate the shear-thinning properties.

**Fourier-Transform Infrared Spectroscopy (FTIR):** An FTIR spectrometer (Frontier, PerkinElmer Inc., Waltham, MA, USA) was used to characterize the chemical bond vibrations and functional groups in MCC, FG, and MCC/FG composite hydrogels. Spectra were recorded in the range of 4000–500 cm^−1^ with a resolution of 4 cm^−1^ and 32 scans per sample.

**X-Ray Diffraction Analysis (XRD):** The crystallographic structures of FG, MCC, and FG/MCC composite powders were characterized using a powder X-ray diffractometer (D8 Advance, Rigaku Corporation, Tokyo, Japan). The operational parameters were set as follows: accelerating voltage 40 kV, current 40 mA, scan rate 6 °/min, and 2θ range 5–60°.

### 4.5. Characterization of Scaffolds

**Morphological characterization:** The freeze-dried scaffolds were cryofractured by immersion in liquid nitrogen for 2 min and sectioned into 4 mm × 4 mm specimens. The samples were sputter-coated with a gold (Au) layer and analyzed using a field-emission scanning electron microscope (JSM-7401F, Hitachi, High-Tech, Tokyo, Japan) at an accelerating voltage of 5 kV.

**Porosity:** The porosity of the scaffold was evaluated by liquid displacement [20]. First, a designated amount of ethanol was added to a graduated cylinder and weighed ($m_{1}$). Next, a scaffold sample ($m_{s}$) was immersed in ethanol after 40 min to ensure saturation. After soaking, ethanol was added to the initial mark and the total mass ($m_{2}$) was recorded. Lastly, the scaffold was removed, and the remaining ethanol with the cylinder was reweighed ($m_{3}$). The procedure was repeated three times to obtain an average value. The porosity was calculated using Equation (3) [21]:

(3) $$P_{o}=\frac{m_{2}-m_{3}-m_{s}}{m_{1}{-m}_{3}}\times 100\%$$

**Equilibrium swelling ratio:** The swelling ratio of the scaffold was quantified gravimetrically. The dried scaffold was immersed in deionized water. The scaffold sample’s dry weight was recorded as $w_{0}$. The sample was periodically weighed until mass stabilization, and the equilibrium swollen weight was denoted as $w_{\infty }$. Triplicate measurements were performed to ensure reproducibility. The equilibrium swelling ratio was calculated using Equation (4) [21]:

(4) $$S_{eq}=\frac{w_{\infty }-w_{0}}{w_{0}}\times 100\%$$

**Degradation ratio:** The scaffold sample’s dry weight was recorded as $w_{0}$. After immersion in PBS solution for 4 weeks, the dried sample’s weight was recorded as $w_{d}$. The procedure was repeated three times to obtain an average value. The degradation ratio was calculated using Equation (5):

(5) $$D_{e}=\frac{w_{0}-w_{d}}{w_{0}}\times 100\%$$

**Compression strength:** A texture analyzer (Model 2450, Keithley Instruments Inc., Solon, OH, USA) was used to conduct compression testing on the scaffold samples. The samples were placed between parallel cylindrical plates and compressed at a constant displacement rate of 3 mm/min until structural failure occurred. The compressive strength was recorded at 50% strain.

## Data Availability

The data presented in this study are openly available in article.

## Supplementary Materials

The following supporting information can be downloaded at: https://www.mdpi.com/article/10.3390/gels11060458/s1, Figure S1: Relative cell activity (* *p* < 0.05; ** *p* < 0.01); Figure S2: Cells growth of NIH 3T3 fibroblasts cultured using extracts of blank control and FM6 for 24 h, 48 h and 72 h (200×).
