# Supplementary material for: A Case Study on Fish Gelatin/Microcrystalline Cellulose Biomaterial Inks for Extrusion-Based Bioprinting
Source: Gels. 2025 Jun 16;11(6):458. doi: 10.3390/gels11060458 (PMC12191509; doi:10.3390/gels11060458)
Supplement: Supplementary file 1 [file gels-11-00458-s001.zip › gels-3671269-supplementary.pdf]

A Case Study on Fish Gelatin/Microcrystalline Cellulose Biomaterial Inks for Extrusion Bioprinting

Yubo Tao [1], Jinbao Du [1], Tong Hu [1], Peng Li [1, 2*], Ling Pan [3], Fangong Kong [1] and Jingfa Zhang [1*]

[1] State Key Laboratory of Green Papermaking and Resource Recycling, Qilu University of Technology (Shandong Academy of Sciences), Jinan 250353, China
[2] Key Laboratory of Bio-based Material Science & Technology, Northeast Forestry University, Harbin 150040, China
[3] School of Art and Design, Wuhan Polytechnic University, Wuhan 43004, China

* Corresponding author. E-mail addresses: lipeng@qlu.edu.cn (P. Li); zjf2010dl@126.com (J. Zhang)

Support information

1. Vitro cytotoxicity assessment

1.1 Materials

Newborn Calf Serum (MP20005-500ml, Source Leaf Biological Co., Ltd.), DMEM (10-013-CVRC, Corning Co., Ltd.), PBS (WH0112201 911XP, Procell Co., Ltd.), Trypsin (143188, Biosharp Co., Ltd.), L-Glutamine (GNM21051, Gino Biotech Co., Ltd.), Sodium Pyruvate (GNM11444, Gino Biotech Co., Ltd.), Non-Essential Amino Acids (GNM71450, Gino Biotech Co., Ltd.), DMSO (Tianjin Fuyu Fine Chemical Co., Ltd.), MTT (M8180, Solarbio Co., Ltd.), Live/Dead Cell Staining Kit (BB-4126, Shanghai BestBio Co., Ltd.), NIH-3T3 cells (iCell Bioscience Shanghai).

1.2 Preparation of MCC/FG Composite

The 6% (w/w) FG/MCC mixed solution was injected into a polytetrafluoroethylene (PTFE) mold and dried at room temperature to obtain the FM composite sample.

1.3 MTT Assay

The cell culture medium was prepared by mixing DMEM high-glucose medium, newborn calf serum, sodium pyruvate, L-glutamine, and non-essential amino acids at a ratio of 87:10:1:1:1 (v/v). NIH3T3 cells were cultured in a 5% CO_2, 37°C incubator, with sterilized FM samples prepared as a 25.6 mg/mL stock solution and diluted to 2, 1, 0.5, and 0.25 mg/mL for testing. The experiment included 5 groups: Control (100 μL/well of complete medium) and five concentration groups (Concentration 1-4), each tested in triplicate (n=3). NIH3T3 cells in logarithmic growth phase were counted and seeded at 3×10^3 cells/well in 96-well plates, cultured for 24, 48, and 72 h (5% CO_2, 37°C) before sample-containing medium removal. For MTT assay, wells were washed 3× with PBS, incubated with 100 μL of 0.5 mg/mL MTT for 4 h

($5\%\ CO_2$, 37°C), then treated with 100 μL DMSO after supernatant removal, with absorbance measured at 570 nm after 10 min shaking.

1.4 Cell Viability Assessment by Live/Dead Staining

The viability of NIH3T3 cells co-cultured with test samples for 24 h was evaluated using Live/Dead staining. The cell culture medium preparation and cell culture conditions followed the same protocol described in Section 1.2. FM samples were prepared as a stock solution (25.6 mg/mL) and diluted to 2 mg/mL for testing. The experiment included two groups: Control (1 mL/well of complete medium) and Group (2 mg/mL sample concentration). NIH3T3 cells in the logarithmic growth phase were counted and seeded into confocal dishes at a density of 6×10^4 cells/dish, followed by treatment according to the designated groups. Cells were cultured in a $5\%\ CO_2$, 37°C incubator for 24 h. For staining, Reagent A (Calcein-AM) and Reagent B (PI) were each diluted 10-fold with Diluent C (Solution C). A working staining solution was freshly prepared by mixing 985.5 μL serum-free medium, 10 μL diluted Reagent A, and 4.5 μL Reagent B. After removing residual serum by washing once with PBS, cells were incubated with 500 μL/well staining solution at room temperature (protected from light) for 15 min before imaging.

1.5 Results and discussion

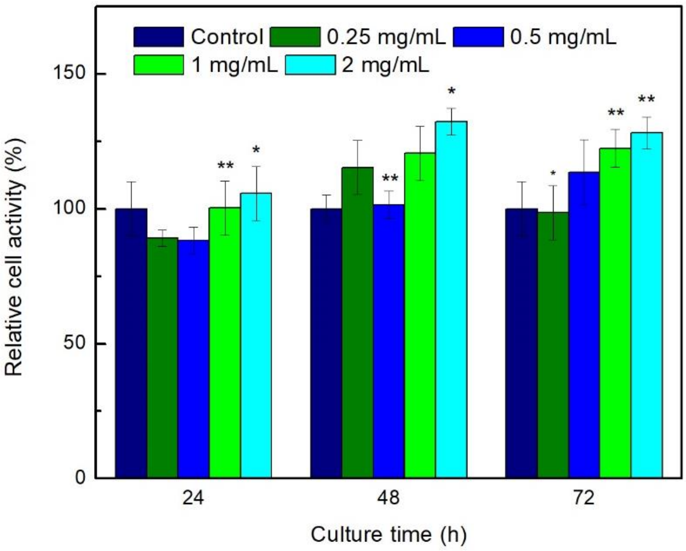

Figure S1. Relative cell activity (*P<0.05；**P<0.01)

The MTT results of sample at different culture times (24 h, 48 h and 72h) are shown in Figure 15. Notably, FM sample exhibited good cytocompatibility and maintained a cell viability greater than 80%. Moreover, cell viability did not diminish over the passage of time. This indicates that the FG/MCC composite had no inhibitory effects on cell growth.

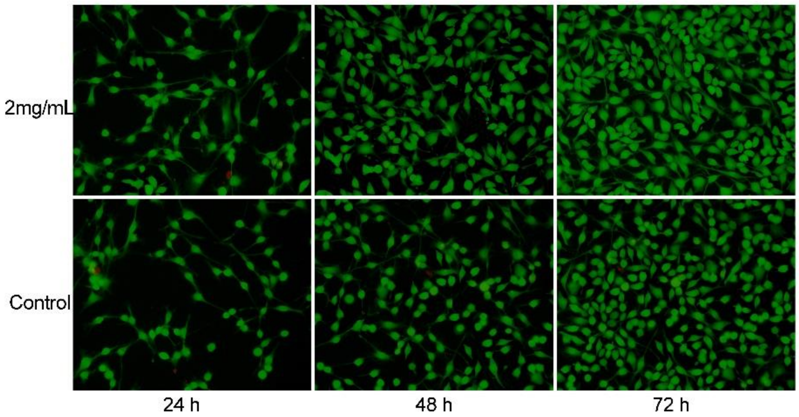

Figure S2 Cells growth of NIH 3T3 fibroblasts cultured using extracts of blank control and FM6 for 24h, 48h and 72 h (200x)

Live/dead assay experiments were conducted to visually examine cell deaths. As depicted in Figure S2, where live cells are strain green, no dead cells (stained red) could be identified among the tested samples. The results from the live/dead assay agree with results from the MTT assay. Hence, it can be concluded that the FG/MCC composite is non-cytotoxic.

2. Viscosity of Commercial Fish Skin Gelatin and Laboratory-Prepared Gelatin

The gelatin solutions were prepared at a concentration of 6.67% (w/w) for both commercial fish skin gelatin (Wuhan Huaxiang Kejie Biotechnology Co., Ltd) and laboratory-prepared gelatin. Viscosity measurements were conducted at 60°C using a viscometer (Model: NDJ-8S, Shanghai Qigao Instrument Co., Ltd.) with a rotational speed of 12 rpm. The results indicated that the viscosity of the laboratory-prepared gelatin ranged from 82 to 84 cP, while the commercial gelatin exhibited a lower viscosity of 53 to 56 cP.
